# Ictal Hypersalivation and Salivary Gland Enlargement in a Patient With Acquired Frontal Lobe Epilepsy

**DOI:** 10.7759/cureus.15319

**Published:** 2021-05-29

**Authors:** Edward C Mader, Xinran M Xiang, Piotr W Olejniczak, Daniella Miller

**Affiliations:** 1 Neurology, Louisiana State University Health Sciences Center, New Orleans, USA; 2 Pediatric Neurology, Oregon Health & Science University, Portland, USA; 3 Pediatric Neurology, Louisiana State University Health Sciences Center, New Orleans, USA

**Keywords:** hypersalivation, drooling, salivary, parotid, seizure, epilepsy, ictal, rolandic, operculum

## Abstract

Hypersalivation is a well-known ictal semiology of benign Rolandic epilepsy and other childhood epilepsy syndromes. There are also occasional reports of adults with temporal, parietal, or frontal lobe epilepsy in which hypersalivation is a prominent seizure manifestation. Notably lacking are reports linking salivary gland enlargement to ictal hypersalivation. A 33-year-old man with frontal lobe epilepsy due to a ruptured aneurysm presented with focal seizures and facial swelling. The only seizures he had in the past were generalized tonic-clonic seizures. Eight days prior to admission, he started having focal seizures characterized by pronounced hypersalivation, speech arrest, impaired awareness, and left upper extremity posturing or automatism. Seizure frequency increased from five to 30 per day. Four days prior to admission, his face started to swell up, and his family thought he had mumps. Computed tomography (CT) of the head showed encephalomalacia in the inferomedial cortex of the right frontal lobe, the same lesion seen in his old CT images. Maxillofacial CT revealed enlargement of the parotid and submandibular glands. Although electroencephalography (EEG) showed seizure onset in the right frontal region, the initial ictal discharge on the scalp may represent seizure propagation from a focus near the zone of encephalomalacia. After seizure freedom was achieved with antiepileptic drugs, the patient’s salivary glands decreased in size and returned to normal.

## Introduction

Saliva flows constantly at a basal rate with gustatory stimuli and chewing triggering intermittent surges in salivation [1]. The major salivary glands, namely, parotid, submandibular, and sublingual, are under autonomic control [2]. Sensory information from the oropharynx is relayed to the salivatory nuclei in the medulla. The facial, glossopharyngeal, and vagus nerves conduct signals from chemoreceptors and the trigeminal nerve conducts signals from mechanoreceptors and nociceptors [1-2]. Both parasympathetic and sympathetic nerves stimulate salivary gland secretion but the effects of parasympathetic stimulation are stronger and longer-lasting [3]. Salivary secretion is influenced by thought and emotion through direct and indirect projections of forebrain structures to brainstem salivatory nuclei [1-2]. Autonomic nerves do not only stimulate salivary gland secretion, but they also play a vital role in salivary gland maintenance, regeneration, and atrophy prevention [4].

Hypersalivation or drooling is due to increased saliva secretion (sialorrhea) from salivary gland overstimulation, decreased saliva clearance from oropharyngeal muscle contraction, or both [5]. Ictal hypersalivation is a prominent feature of classic benign childhood epilepsy with centrotemporal spikes (BCECTS) a.k.a. benign Rolandic epilepsy [6]. Hypersalivation can also be a salient ictal semiology in BCECTS with atypical features [7], Panayiotopoulos syndrome [8], and other benign childhood seizure susceptibility syndromes [9]. Drooling during a seizure is not uncommon in adults, but hypersalivation as the dominant ictal semiology has been reported in only a few cases of acquired temporal, parietal, and frontal lobe epilepsy. We present an adult patient with acquired frontal lobe epilepsy in whom frequent ictal hypersalivation resulted in enlargement of the salivary glands.

## Case presentation

A 33-year-old African-American man with type-1 diabetes mellitus, hypertension, and frontal lobe epilepsy presented with focal seizures and facial swelling. He started having generalized tonic-clonic seizures (GTCS) at the age of 31, about a year after he had a right frontal hemorrhage from an aneurysmal rupture. GTCS occurred every three to four months. He took levetiracetam 750 mg bid for seizures. Other medications include insulin, amlodipine, lisinopril, hydralazine, hydrochlorothiazide, metoprolol, aspirin, and lovastatin. He only had GTCS in the past until eight days prior to admission, when he started having focal seizures. The focal seizures consisted of two-minute episodes of drooling, speech arrest, impaired awareness, and left upper extremity posturing or automatism, usually scratching of the face. Seizure frequency increased gradually from five to 30 per day over an eight-day period. Four days prior to admission, he developed facial swelling on the right side below and in front of the ear. The next day, the same area on the left side also swelled up. The swelling became increasingly painful and started to interfere with speech and swallowing. His family thought he had mumps.

**Figure 1:**
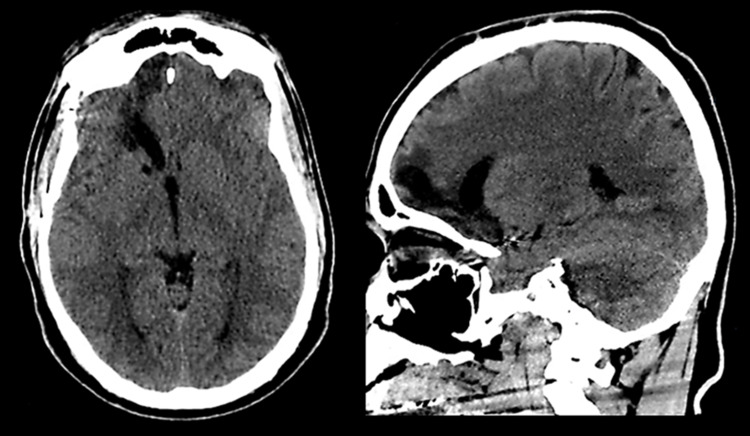
Head CT showing right inferomedial frontal encephalomalacia and evidence of past aneurysm surgery The right anterior encephalomalacia correlates with the site of parenchymal injury due to rupture of a communicating artery aneurysm three years prior to admission. The aneurysm was clipped. Previous CT images showed the same findings.

**Figure 2:**
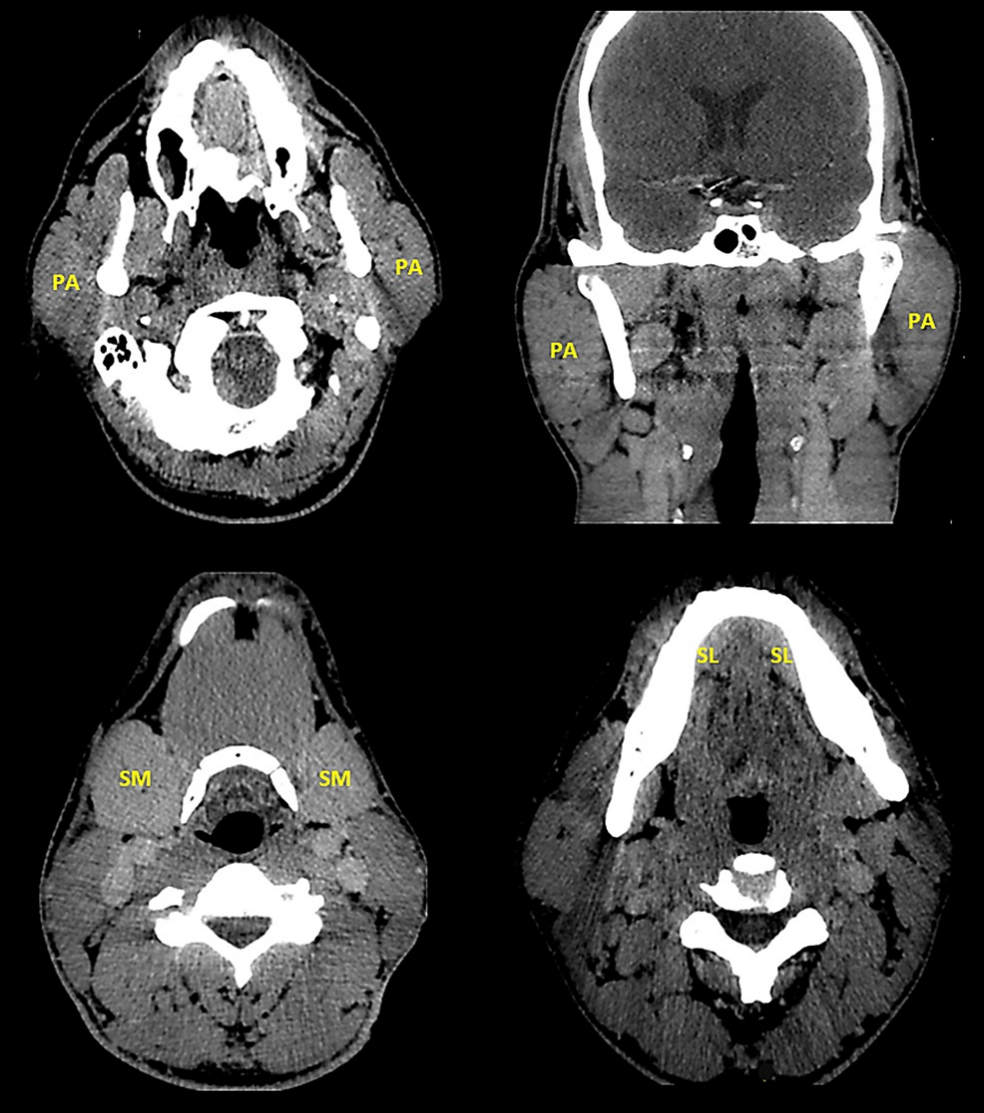
Maxillofacial CT showing enlargement of the parotid glands (PA) and submandibular glands (SM). The sublingual glands (SL) are either normal or slightly enlarged. Parotid gland swelling is more pronounced than submandibular gland swelling and, for both glands, swelling is more pronounced on the right side.

On admission, he had bifacial swelling that was more pronounced on the right side. He was attentive and fully oriented but had difficulty speaking because of the swelling. Blood pressure was 125/65 mmHg, pulse rate 72/min, respiratory rate 15/min, and oxygen saturation 98.3%. His temperature was 37.1 °C and meningeal signs were absent. Neurological exam showed intact cranial nerves, 5/5 muscle strength, symmetric 1+ reflexes, intact cerebellar function, normal sensation, and stable posture and gait. Blood counts and chemistries were normal, except for blood glucose (initially 325 mg/dL but later decreased to 152 mg/dL). Head computed tomography (CT) showed right inferomedial frontal encephalomalacia and evidence of past aneurysm surgery (Figure 1). Otolaryngology was consulted. Maxillofacial CT showed enlargement of the parotid and submandibular glands (Figure 2). Parotid gland swelling was more pronounced than submandibular gland swelling and, for both glands, the swelling was more prominent on the right side. Clindamycin and cefazolin were started but discontinued after four days based on the recommendation of the otolaryngologist, who did a thorough examination of the patient's oral cavity and found no local signs of infection. Cultures of saliva samples and blood were also negative.

**Figure 3:**
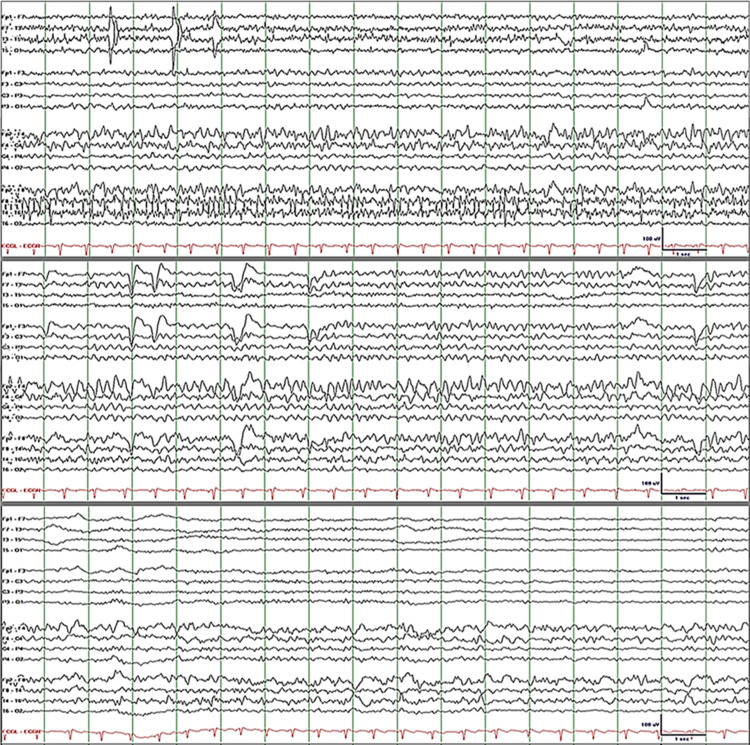
EEG recorded during a seizure showing right frontal (F4) rhythmic alpha activity at seizure onset (top tracing) followed by evolutionary changes in frequency (alpha to theta with superimposed delta) as the seizure recruits neighboring and left hemisphere circuits (middle tracing). Interictal EEG shows right temporal and frontal polymorphic delta activity and breach rhythm due to skull defect (bottom tracing). Display parameters: digital filter bandpass of 1-70 Hz with 60-Hz notch filter turned on; voltage-time scale at the lower right corner; longitudinal bipolar montage with channels grouped from top to bottom as follows: left temporal, left parasagittal, right parasagittal, right temporal, and EKG. EEG: electroencephalography; EKG: electrocardiogram

**Figure 4:**
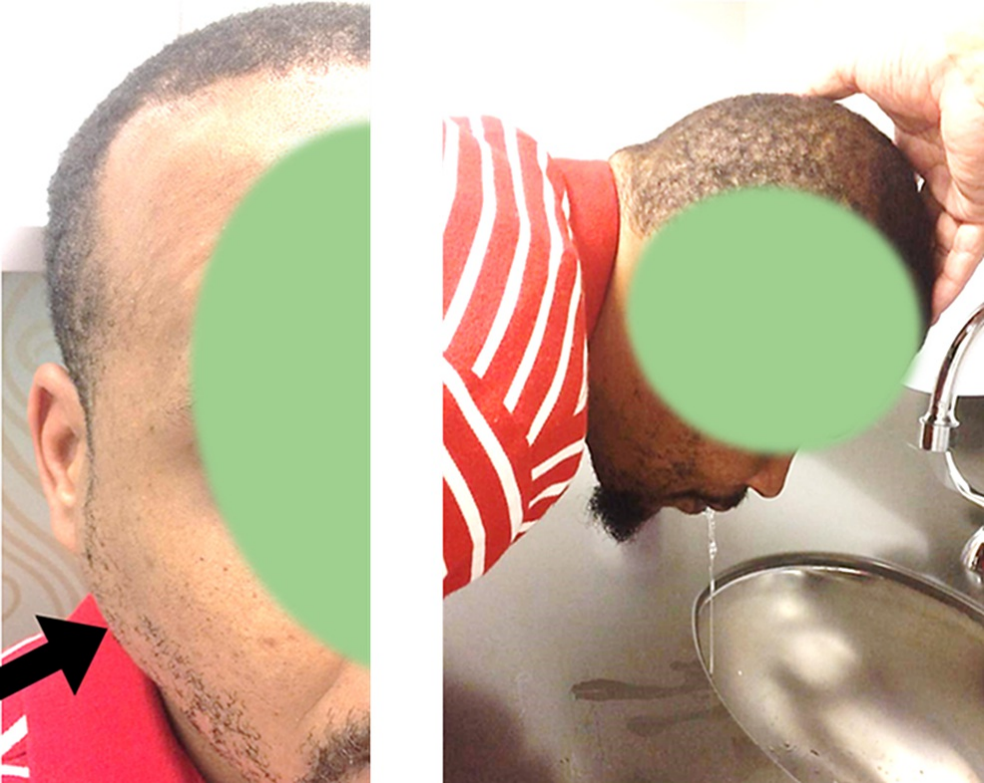
Photos taken during his clinic visit two months after discharge He complained of increasing seizure frequency (from 3 to 50 per day) and recurrence of facial swelling. Parotid gland enlargement is shown on the left (arrow) and ictal hypersalivation on the right.

Electroencephalography (EEG) revealed a seizure with onset in the right frontal area (F4) followed by regional and contralateral spread (Figure 3). He had drooling, speech arrest, impaired awareness, and left arm posturing during the seizure. Levetiracetam 2000 mg intravenous (IV) was loaded, followed by 1000-mg IV q12h. Lacosamide 50 mg IV q12h was also started. Seizure frequency decreased from 30 per day on Day 1 to three per day on Day 8. Daily physical examination showed a gradual decrease in parotid gland swelling and tenderness over an eight-day period. The patient's ability to speak and swallow improved accordingly. The physicians saw no reason to repeat the maxillofacial CT. On Day 8, he was discharged on levetiracetam 1000 mg bid and lacosamide 50 mg bid. Two months after discharge, he came to the clinic and informed us that his seizures have become more frequent (from 3 to 50 per day) and his face has swelled up again. Just like in the past, the swelling was more prominent on the right side of the face (Figure 4: Left). He had a seizure while he was in the clinic (Figure 4: Right). Levetiracetam was increased to 1500 mg bid and lacosamide was increased to 100 mg bid. Three months later, he returned to the clinic and informed us that he was seizure-free for more than two months. His face was no longer swollen, and he no longer had difficulty speaking and swallowing.

## Discussion

Pure salivatory seizures are exceptionally rare. More often than not, ictal hypersalivation is accompanied by other ictal manifestations. In BCECTS, ictal hypersalivation is often associated with speech arrest, dysarthria, dysphagia, or lingual apraxia [6]. Fogarasi et al. detected autonomic symptoms in a large percentage of childhood focal seizures; hypersalivation was one of the ictal symptoms in 5% of the patients [10]. Gayatri et al. reported two children with persistent drooling, dysarthria, and dysphagia that resolved completely with empiric antiepileptic therapy [11]. Prosepio et al. evaluated nocturnal complex motor seizures with stereotactic EEG in eight patients and detected visceral/autonomic symptoms, including hypersalivation, dysarthria, and apnea, during ictal activation of the insular and opercular cortex [12]. In 1954, Penfield and Jasper reported a case of pure salivatory seizures [13]. The patient became seizure-free after surgical removal of a cyst in the right postcentral gyrus. Recently, Nascimento et al. reported pure salivatory seizures in an adolescent with subtle cortical malformation of the right parietal cortex [14]. 

Several cortical structures have been implicated in the pathophysiology of ictal hypersalivation. In patients with BCECTS, where hypersalivation is a prominent ictal manifestation, the epileptogenic zone, as well as the irritative zone, appears to be located in the Rolandic cortex, particularly the lower Rolandic area [15-17]. Ictal hypersalivation has also been associated with lesions in the peri-Sylvian area or in the mesial temporal lobe [18-20]. It is also worth mentioning that hypersalivation can be the expression not of seizure initiation but of seizure propagation. By performing stereotactic EEG, Prosepio et al. detected simultaneous ictal discharges in the insula and operculum during ictal hypersalivation [12]. Morita et al. attributed ictal hypersalivation in temporal lobe epilepsy to seizure propagation from the hippocampus to the frontal limbic cortex [19]. Without intracranial EEG data, we can only speculate that our patient’s seizures originated from epileptic networks near the site of injury in the right inferomedial frontal cortex. Ictal hypersalivation, left upper extremity motor phenomena, and impaired awareness suggest seizure propagation from the inferomedial frontal cortex to the frontal operculum, the right motor cortex, and the limbic cortices, respectively. Likewise, the EEG seizure onset on the scalp at F4 may represent seizure propagation from a deep frontal lobe focus to a more superficial frontal lobe cortex.

We are not aware of any publication wherein salivary gland enlargement was specifically reported as a complication of ictal hypersalivation. Apparently, the parotid and submandibular glands of our patient increased in size because of frequent ictal stimulation. Salivary gland size depends on the trophic effects of nerves and on glandular stimulation [1,5]. Parasympathetic innervation is of particular importance for maintaining gland size and secretory capacity [1,3]. Animal studies have shown that gland size, secretory capacity, and neuronal acetylcholine synthesis decrease as the demand for salivary secretion decreases (e.g. liquid diet) and increase as the demand for salivary secretion increases (e.g. chewing-demanding diet) [1]. Despite prominent ictal hypersalivation, salivary gland enlargement is not a salient feature of BCECTS perhaps because seizures do not occur frequently in this epilepsy syndrome.

## Conclusions

Ictal hypersalivation is prominent in some childhood epilepsy syndromes, notably benign Rolandic epilepsy. Drooling during a seizure is not uncommon in adults, but hypersalivation as a prominent ictal semiology has been reported in only a few cases of acquired temporal, parietal, and frontal lobe epilepsy. This case is unique in that frequent stimulation of the salivary glands during frontal lobe seizures resulted in salivary gland enlargement. Seizure frequency and salivary gland swelling were significantly reduced by increasing the dose of levetiracetam and adding lacosamide. However, seizure frequency and salivary gland swelling increased again. When seizure freedom was finally achieved with higher doses of levetiracetam and lacosamide, the salivary glands returned to their normal size and did not swell up again.
